# Role of Pannexin 1 ATP-Permeable Channels in the Regulation of Signaling Pathways during Skeletal Muscle Unloading

**DOI:** 10.3390/ijms221910444

**Published:** 2021-09-28

**Authors:** Ksenia A. Zaripova, Ekaterina P. Kalashnikova, Svetlana P. Belova, Tatiana Y. Kostrominova, Boris S. Shenkman, Tatiana L. Nemirovskaya

**Affiliations:** 1 Institute of Biomedical Problems, RAS, 123007 Moscow, Russia; katsu.no.himitsu@gmail.com (K.A.Z.); mochalova_ekaterina@lenta.ru (E.P.K.); swetbell@mail.ru (S.P.B.); bshenkman@mail.ru (B.S.S.); 2 Department of Anatomy, Cell Biology and Physiology, Indiana University School of Medicine-Northwest, Gary, IN 46408, USA; tkostrom@iun.edu

**Keywords:** muscle unloading, pannexin channel 1, MuRF1, MAFbx

## Abstract

Skeletal muscle unloading results in atrophy. We hypothesized that pannexin 1 ATP-permeable channel (PANX1) is involved in the response of muscle to unloading. We tested this hypothesis by blocking PANX1, which regulates efflux of ATP from the cytoplasm. Rats were divided into six groups (eight rats each): non-treated control for 1 and 3 days of the experiments (1C and 3C, respectively), 1 and 3 days of hindlimb suspension (HS) with placebo (1H and 3H, respectively), and 1 and 3 days of HS with PANX1 inhibitor probenecid (PRB; 1HP and 3HP, respectively). When compared with 3C group there was a significant increase in ATP in soleus muscle of 3H and 3HP groups (32 and 51%, respectively, *p* < 0.05). When compared with 3H group, 3HP group had: (1) lower mRNA expression of E3 ligases MuRF1 and MAFbx (by 50 and 38% respectively, *p* < 0.05) and MYOG (by 34%, *p* < 0.05); (2) higher phosphorylation of p70S6k and p90RSK (by 51 and 35% respectively, *p* < 0.05); (3) lower levels of phosphorylated eEF2 (by 157%, *p* < 0.05); (4) higher level of phosphorylated GSK3β (by 189%, *p* < 0.05). In conclusion, PANX1 ATP-permeable channels are involved in the regulation of muscle atrophic processes by modulating expression of E3 ligases, and protein translation and elongation processes during unloading.

## 1. Introduction

During hypokinesia, skeletal muscle undergoes atrophy due to the disbalance between protein synthesis and protein degradation [1,2,3]. Initial changes in signaling pathways occur within minutes/hours after unloading [4,5]. Nevertheless, physiological mechanisms that regulate these processes are not completely understood. It was previously reported that ten days of muscle unloading leads to the accumulation of high-energy phosphates (PCr) [6] and calcium ions [7,8,9] in muscle fibers. At the same time, in a different study it was shown that at fourteen days of unloading ATP and PCr content are decreased [10]. Pharmacologically-induced decrease in the levels of high-energy phosphates and calcium ions in unloaded skeletal muscle results in the decrease of both muscle atrophy and slow-to-fast fiber type switching in soleus muscle [6,9,11,12]. Moreover, previous studies showed that extracellular ATP is one of the major autocrine-paracrine regulators of cell signaling activated in response to diverse stimuli, including hormones, neurotransmitters, mechanical stimuli, and inflammation [13]. Extracellular ATP might be one of the major regulators of physiological processes activated in unloaded skeletal muscle, including changes in the gene expression patterns.

The release of ATP into extracellular space is regulated via dihydropteridine receptor (DHPR) and PANX1 channels [14]. We hypothesized that muscle unloading changes plasma membrane potential and leads to the activation of Ca-dependent DHPR channels located in close proximity to the PANX1 channels. Pannexins were first reported in 2000 as a new family of proteins capable to form membrane channels [15]. The pannexin family has three members (PANX1, PANX2, and PANX3) [16]. PANX1 is ubiquitously expressed while PANX2 and PANX3 have more limited expression pattern. The predominant isoform in skeletal muscle is PANX1 [17]. This is a 48 kilodalton protein that allows transport of ATP from sarcoplasm into extracellular space [16,18,19]. *Panx2* and *Panx3* are expressed only at very low RNA levels without verifiable protein expression (https://www.proteinatlas.org; accessed on 9 September 2021). We previously reported that inhibition of DHPR and decrease of high-energy phosphates diminishes unloading-induced muscle atrophy and metabolic changes [9,11,12]. Physiological mechanisms of these effects were not fully elucidated in our previous studies.

In our current model, three days of muscle unloading promotes the release of ATP and other nucleotides from the sarcoplasm into extracellular space and can facilitate their interaction with the P2Y channels. Activation of P2Y channels leads to the activation of PI3K and subsequently to the stimulation of IP3 receptors located in the nuclei and sarcoplasmic reticulum. Previous studies showed that agonist-activated purinergic receptors promoted the release of calcium ions via IP3-dependent and RyR-independent mechanisms [20]. Casas and colleagues [14] reviewed interactions of DHPR, PANX1, G proteins, PLC, and PI3K in the sarcolemma. Dr. Jaimovich’s laboratory at the University of Chile investigated role of PI3K in this signaling pathway [21,22,23].

The goal of the current study is to assess the role of high-energy phosphates and their transport via PANX1 channels in the regulation of unloading-induced signaling pathways. Using an inhibitor of PANX 1, probenecid (PRB), during one and three days of hindlimb suspension, we evaluated how unloading-induced increase of ATP affects signaling pathways regulating protein synthesis and degradation. If our hypothesis is correct, inhibition of PANX1 during unloading will result in the significant changes of the key players in signaling pathways.

## 2. Results

### 2.1. Effect of Probenecid on the Unloaded Soleus Muscle Weight, Panx1 Expression and ATP Content

After one day of unloading there were no significant changes in soleus muscle weight between 1C, 1H, and 1HP groups (Table 1). After three days of unloading, soleus muscle weight was significantly lower in both 3H and 3HP groups (by 16 and 18%, respectively, *p* < 0.05) when compared with 3C (Table 1).

**Figure 1:**
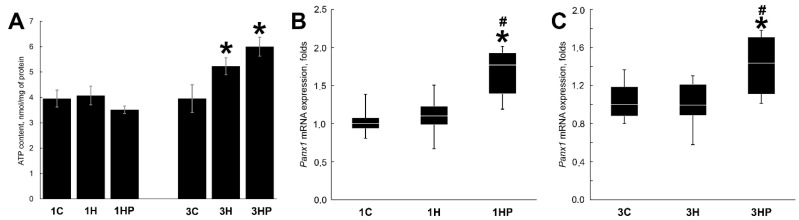
Evaluation of the ATP content (**A**) and *Panx1* mRNA expression (**B**,**C**) in soleus muscles of non-treated control rats (1C and 3C), rats after 1 and 3 days of unloading (1HS and 3HS), and 1 and 3 days of HS with PRB inhibitor (1HP and 3HP). Levels of *Panx1* mRNA were normalized to the levels of *Gapdh* in each sample (**B**,**C**). n = 8. * indicates a significant difference from the control, ^#^ indicates a significant difference from the HS group, *p* < 0.05.

There were no significant differences in the ATP content between 1C, 1H, and 1HP groups after one day of unloading (Figure 1A). At three days of unloading ATP content of soleus muscle increased by 32% (3H group, *p* < 0.05). Inhibition of PANX1 with PRB resulted in further increase of ATP content (by 19%, 3HP group, *p* < 0.05; Figure 1A).

Expression of *Panx1* was not changed after one and three days of unloading (Figure 1B,C). Inhibition of PANX1 with PRB resulted in the significant increase of *Panx1* mRNA expression in both 1HP and 3HP groups (by 77 and 44%, respectively, *p* < 0.05) when compared with C and H groups (Figure 1B,C).

### 2.2. Effect of Probenecid on the Catabolic Processes in Unloaded Soleus Muscle

**Figure 2:**
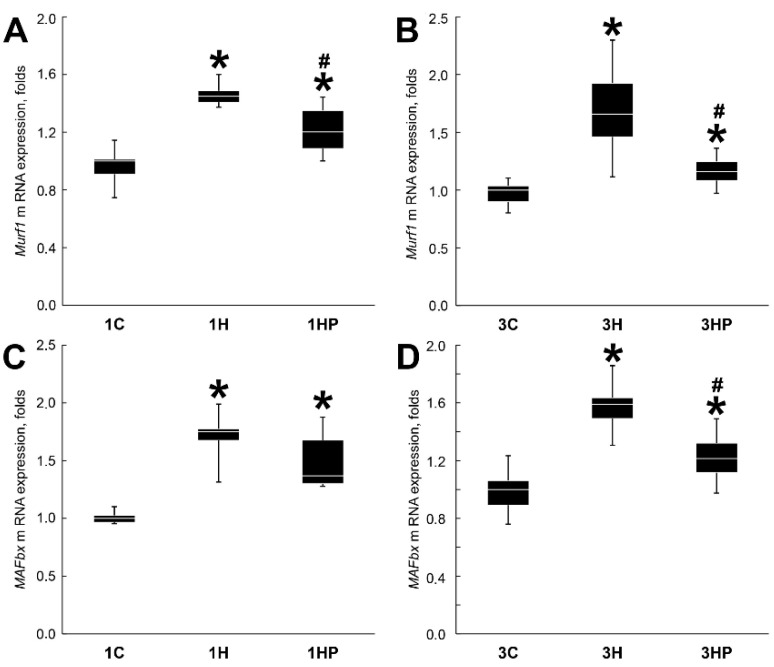
Evaluation of the *MuRF1* (**A**,**B**) and *MAFbx* (**C**,**D**) mRNA expression in soleus muscles of non-treated control rats (1C and 3C), rats after 1 and 3 days of unloading (1HS and 3HS), and 1 and 3 days of HS with PRB inhibitor (1HP and 3HP). Levels of *MuRF1* and *MAFbx* mRNA were normalized to the levels of *Gapdh* in each sample. n = 8. * indicates a significant difference from the control, ^#^ indicates a significant difference from the HS group, *p* < 0.05.

After one and three days of unloading the expression of *MAFbx* and *MuRF1* in soleus muscle was significantly increased (Figure 2). When compared with 1H and 3H groups, *MuRF1* mRNA expression was diminished in both 1HP and 3HP groups (by 25 and 50%, respectively, *p* < 0.05; Figure 2AB). Expression of *MAFbx* mRNA was significantly reduced only in 3HP group (by 38%, *p* < 0.05; Figure 2D) when compared with 3H group.

**Figure 3:**
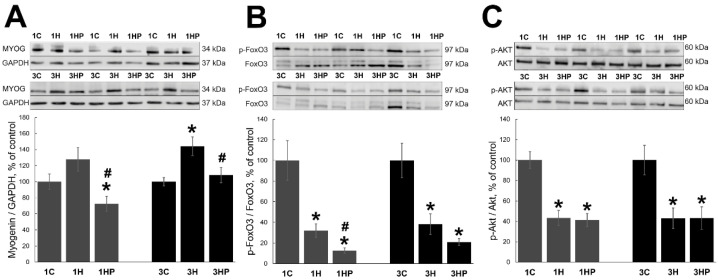
Evaluation of the MYOG (**A**), p-FoxO3 (**B**) and p-AKT (**C**) content in soleus muscles of non-treated control rats (1C and 3C), rats after 1 and 3 days of unloading (1HS and 3HS), and 1 and 3 days of HS with PRB inhibitor (1HP and 3HP). Values are normalized to the level of GAPDH (**A**), total FoxO3 (**B**) and total Akt (**C**) in each sample. n = 8. * indicates a significant difference from the control, ^#^ indicates a significant difference from the HS group, *p* < 0.05.

Expression of *MAFbx* and *MuRF1* is regulated by the transcription factors MYOG [24,25,26] and FOXO3 [27,28]. Unloading resulted in increased expression of MYOG in 3H group (by 44%, *p* < 0.05; Figure 3A) when compared with 3C group. There was a trend for the increase of MYOG expression in 1H group, but the values did not reach statistically significant levels (Figure 3A). Inhibition of PANX1 with PRB blocked unloading-induced MYOG upregulation in 1HP and 3HP groups (*p* < 0.05). In fact, MYOG expression in 1HP group was significantly lower than in 1C group (*p* < 0.05; Figure 3A).

Transcriptional activity of FoxO3 is regulated by phosphorylation [29]. AKT can regulate FoxO3 phosphorylation at Thr32, Ser253 and Ser315 sites [30]. Unloading resulted in significant decrease of phospho-FoxO3 (p-FoxO3) in both 1H and 3H groups (by 7% and 58%, respectively, *p* < 0.05; Figure 3B) when compared with 1C and 3C groups. PRB treatment has not prevented the decrease of p-FoxO3. In fact, FoxO3 phosphorylation was lower in both 1HP and 3HP groups when compares with 1H and 3H groups (Figure 3B).

Similarly, phospho-AKT (p-AKT) content was significantly decreased in all four groups with soleus muscle unloading (Figure 3C).

### 2.3. Effect of Probenecid on the Anabolic Processes in Unloaded Soleus Muscle

**Figure 4:**
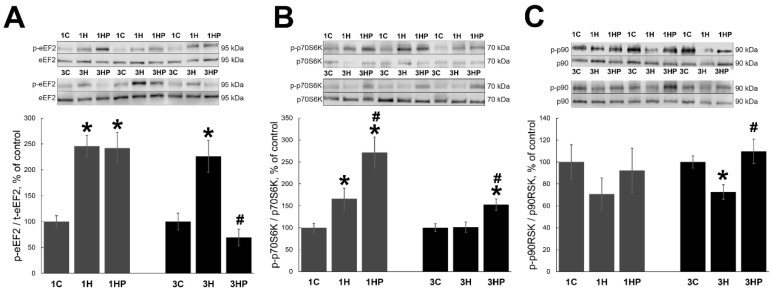
Evaluation of the p-eEF2 (**A**), p-p70S6K (**B**) and p-p90RSK (**C**) content in soleus muscles of non-treated control rats (1C and 3C), rats after 1 and 3 days of unloading (1HS and 3HS), and 1 and 3 days of HS with PRB inhibitor (1HP and 3HP). Values are normalized to the level of total eEF2 (**A**), total p70S6K (**B**) and total p90RSK (**C**) in each sample. n = 8. * indicates a significant difference from the control, ^#^ indicates a significant difference from the HS group, *p* < 0.05.

eEF2 is a key regulator of protein translation [31]. Unloading resulted in a significant increase of phospho-eEF2 (p-eEF2) in both 1H and 3H groups (by 146% and 126%, respectively, *p* < 0.05; Figure 4A) when compared with 1C and 3C groups. Inhibition of PANX1 with PRB had no effect on the p-eEF2 content at one day of unloading but decreased unloading-induced eEF2 phosphorylation in 3HP group (by 157%, *p* < 0.05; Figure 4A) when compared with 3H group. In fact, eEF2 phosphorylation in 3HP group was lower than in 3C group (Figure 4A).

p70S6K and p90RSK regulate skeletal muscle protein synthesis [1]. One day of unloading increased the content of phospho-p70S6K (p-p70S6K; by 66% in group 1H, *p* < 0.05; Figure 4B) when compared with 1C group. At three days of unloading the content of p-p70S6K in unloaded muscle was not different from the control (Figure 4B). Inhibition of PANX1 with PRB resulted in a significant increase of p-p70S6K content in both 1HP and 3HP groups (by 172% and 53%, respectively, *p* < 0.05; Figure 4B) when compared with 1C and 3C groups.

At one day of unloading, phospho-p90RSK (p-p90RSK) content showed a decreasing trend without reaching statistically significant values in both 1H and 1HP groups when compared with 1C group (Figure 4C). When compared with the control group, three days of unloading resulted in the significant decrease of p-p90RSK content in 3H group (by 27%, *p* < 0.05) while in 3HP group it was similar to the control value (Figure 4C).

### 2.4. Effect of Probenecid on the Signaling Cascades Regulating Homeostasis of Unloaded Soleus Muscle

**Figure 5:**
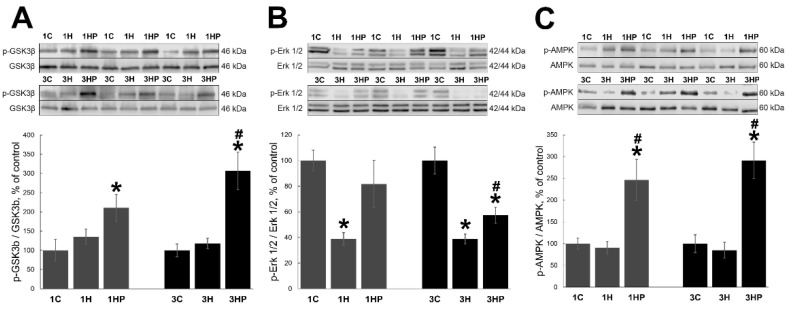
Evaluation of the p-GSK3b (**A**), p-ERK1/2 (**B**) and p-AMPK (**C**) content in soleus muscles of non-treated control rats (1C and 3C), rats after 1 and 3 days of unloading (1HS and 3HS), and 1 and 3 days of HS with PRB inhibitor (1HP and 3HP). Values are normalized to the level of total GSK3b (**A**), total ERK1/2 (**B**) and total AMPK (**C**) in each sample. n = 8. * indicates a significant difference from the control, ^#^ indicates a significant difference from the HS group, *p* < 0.05.

We evaluated signaling cascades regulating muscle homeostasis and affecting muscle atrophy: GSK-3β, ERK1/2, and AMPK. Unloading had no effect on the level of phospho-pGSK-3β (p-GSK-3β) in soleus muscle (1H and 3H groups) when compared with the control (Figure 5A). Treatment with PRB resulted in significant increase of p-GSK-3β content in both 1HP and 3HP groups (by 111% and 206%, respectively, *p* < 0.05) when compared with 1C and 3C groups (Figure 5A).

Phospho-Erk1/2 (p-Erk1/2) content was decreased after muscle unloading (1H and 3H groups) when compared with the control (Figure 5B). Treatment with PRB diminished unloading-induced p-Erk1/2 changes in soleus muscle (Figure 5B). In 1HP group p-Erk1/2 content was not different from the control (1C), and in 3HP group it was lower than in the control (3C) but significantly higher than in the 3H group (Figure 5B).

Unloading had not produced any changes in the phospho-AMPK (p-AMPK) content in soleus muscle (1H and 3H groups; Figure 5C). Treatment with PRB significantly increased p-AMPK content in both 1HP and 3HP groups (by 146% and 191%, respectively, *p* < 0.05) when compared with the control (Figure 5C).

## 3. Discussion

ATP is one of the crucial signaling molecules and it regulates diverse cellular processes. ATP can be an important regulator of skeletal muscle adaptation to unloading. We previously showed that skeletal muscle atrophy and metabolic changes induced by unloading were diminished by the decrease of high-energy phosphate levels [9,11,12]. Nevertheless, the mechanism of this regulation was not explored.

In the current study we showed that ATP content was increased in skeletal muscle after three days of unloading in both PANX1 inhibitor-treated and in untreated rats. Furthermore, PANX1 inhibition with PRB resulted in nineteen percent increase of ATP content in the unloaded muscle. It was previously implied that ATP is released via PANX1 channels [14]. *Panx1* overexpression in rat myoblast L6 cell line resulted in increased extracellular ATP levels at rest and after electrical stimulation [32]. When PRB was used for blocking PANX1 it was suppressing ATP release [33]. We suggest that the increased *Panx1* mRNA expression detected in our study is a compensatory reaction to the functional blockade of PANX1 activity in unloaded muscle.

In our study, PANX1 inhibition had no effect on the decrease of muscle weight in response to unloading. In both PRB-treated and -untreated rats the weight of soleus muscle was decreased by a similar degree. Unloading activates muscle atrophy via upregulation of skeletal muscle-specific E3 ubiquitin ligases MAFbx and MuRF1, as well as some other pathways [2,34]. Nevertheless, sometimes there are discrepancies between the degree of muscle atrophy and the level of expression of E3 ligases [3]. Inhibition of PANX1 with PRB in the current study significantly reduced the upregulation of both MAFbx and MuRF1. We hypothesize that the upregulation of MAFbx and MuRF1 expression after unloading can be associated with the activation of purinergic receptors by ATP. It is known that purinergic receptors have different sensitivity to ATP, ADP, AMP and adenosine [35]. We propose that in our model ATP is released from unloaded muscle fibers into extracellular space via PANX1. Binding of ATP to P2Y receptors can activate signaling cascades and expression of genes regulating unloading-induced muscle atrophy. Our experiments for the first time showed that blocking PANX1 with PRB during unloading results in the reduced expression of E3 ubiquitin ligases.

Several transcription factors regulate MAFbx and MuRF1 expression during unloading [2]. Their activity is controlled by diverse signaling cascades. We tested the role of two such factors: FoxO3 and MYOG. Expression of MYOG is increased in muscle after prolonged immobilization [36] and unloading [37]. MYOG can directly interact with MAFbx and MuRF1 promoters [24,25]. In our study, the level of MYOG expression was increased in muscle after unloading. It was significantly reduced in unloaded muscle of PRB-treated rats. This decrease in MYOG expression could have contributed to the reduced expression of the E3 ubiquitin ligases in muscle of PRB-treated rats.

Some studies indicate that transcription factor FoxO3 can interact with the promoters of MAFbx [2,27,28] and MuRF1 [2] and regulate their expression. In our study, FoxO3 phosphorylation was significantly reduced in unloaded muscle. The decrease in FoxO3 phosphorylation was even more pronounced in the muscle of PRB-treated rats. Therefore, it is very unlikely that FoxO3 contributes to the diminished upregulation of MAFbx and MuRF1 observed in the unloaded muscle of PRB-treated rats. Based on the results of the current study it can be suggested that MYOG is the transcription factor that significantly contributes to the downregulation of MAFbx and MuRF1 expression in unloaded muscle of PRB-treated rats.

AKT phosphorylates FoxO3 on Thr32, Ser203 and Ser315 causing its retention in the cytoplasm and blocking nuclear translocation [30]. In the current study AKT phosphorylation was significantly reduced in unloaded muscle of rats with and without PRB treatment. This correlates with the reduced phosphorylation of FoxO3 in the same samples, suggesting that AKT can be involved in the regulation FoxO3 phosphorylation described in the current study. It has to be mentioned that total AKT and FoxO3 protein levels were upregulated in denervated mouse soleus muscle, while AKT and FoxO3 phosphorylation was not changed [38].

We showed that after one day of unloading the content of p-eEF2 was increased in muscle of both PRB-treated and untreated rats. This finding is in line with the previous studies reporting that the content of p-eEF2 is increased in unloaded skeletal muscle [39,40,41]. At three days of unloading the level of p-eEF2 in muscle of untreated rats was elevated, while the content of p-eEF2 in muscle of PRB-treated rats was similar to the control values. At the same time, the content of p-p70S6K and p-p90RSK in muscle of PRB-treated rats at three days of unloading was elevated. p-p70S6K and p-p90RSK can regulate activity of eEF2 kinase (eEF2K). p-p70S6K and p-p90RSK phosphorylate eEF2K on Ser366 and inhibit its activity [42]. This can result in the dephosphorylation of eEF2 and increased process of translation elongation. Therefore, the inhibition of PANX1 with PRB restores elongation processes in the unloaded muscle via eEF2 kinase-dependent pathway.

GSK-3β performs numerous functions in the cells [43]. In the current study p-GSK-3β content was not changed by unloading. At the same time, unloaded muscle of PRB- treated rats had significantly increased levels of p-GSK-3β. It is proposed that GSK-3β can interact with more than a hundred of different proteins and can phosphorylate many of these proteins. GSK-3β-dependent protein phosphorylation can lead to their ubiquitination via β-TrCP [44]. Decreased PI3K/AKT signaling leads to GSK-3β-mediated desmin phosphorylation, degradation of myofibrils and muscle atrophy [45,46]. Phosphorylation of GSK-3β on Ser9 by AKT inhibits its activity [47,48] and significantly decreases accessibility of Ser9 site. The content of p-AKT was equally decreased in muscle of all unloaded groups. Therefore, a different kinase was mediating increased phosphorylation of GSK-3β in the unloaded muscle of PRB treated rats. GSK-3β phosphorylation can be regulated by cGMP-dependent kinase [49], as well as by PKC, PKA, Rho, cdc2, and CaMKII [50]. It is conceivable that some of these kinases can regulate GSK-3β phosphorylation in the unloaded muscle of PRB-treated rats.

GSK-3β-regulated effects are mediated via TSC2/mTOR pathway [51]. The mTORC1 pathway regulates protein synthesis via p70S6K phosphorylation [1]. High levels of GSK-3β phosphorylation were correlated with increased p70S6K phosphorylation in unloaded muscle of PRB-treated rats in our study. Previous studies showed that p70S6K phosphorylation was increased in muscle after one day of unloading [5,41,52] but returned to control values by the third day. High content of p-p70S6K in unloaded muscle of PRB- treated rats at both one and three days of unloading in our study indicates possible association between the activity of PANX1 and indicators of protein synthesis. Increased p70S6K phosphorylation in unloaded muscle of PRB-treated rats can be related to the phosphorylation and inhibition of GSK-3β.

p90RSK regulates mTOR activity and protein synthesis via phosphorylation of TSC2 and Raptor [53,54]. Protein synthesis can be also affected by the ERK1/2 pathway [1]. ERK1/2 can phosphorylate and activate p90RSK with subsequent phosphorylation of multiple cellular substrates [55,56]. In particular, p90RSK phosphorylation can regulate the activity of GSK-3β [49]. We reported previously that p90RSK phosphorylation was decreased after three days of muscle unloading [39]. In the current study we also showed that p90RSK phosphorylation was decreased after unloading. Inhibition of PANX1 in unloaded muscle of PRB-treated rats attenuated the decline of p90RSK phosphorylation at both one and three days of unloading. ERK1/2 phosphorylation was also decreased by muscle unloading. PRB treatment significantly diminished the unloading-induced decline of ERK1/2 phosphorylation. It can be suggested that regulation of the key anabolic markers during muscle unloading is affected by the PANX1-mediated transport of high-energy phosphates.

AMPK is a key regulator of energy homeostasis. The level of AMPK phosphorylation may vary with the duration of muscle unloading [41,57,58]. In the current study the content of p-AMPK was not significantly changed by unloading. PRB treatment increased AMPK phosphorylation in unloaded muscle at both one and three days of unloading. AMPK phosphorylation may be regulated by several mechanisms, including methylation. It was recently reported that CARM1 can methylate AMPK during early stages of muscle unloading and, as a result, reduce AMPK phosphorylation [59]. We suggest that CARM1 can be involved in the regulation AMPK phosphorylation in the unloaded muscle of PRB-treated rats, but this requires further studies. AMPK activation is regulated by calcium [60]. There might be an association between inhibition of PANX1 with PRB, changes in calcium concentration and the increased phosphorylation of AMPK.

In brief, inhibition of pannexin channels during one or three days of skeletal muscle unloading results in: (1) decreased activity of catabolic signaling, including decreased mRNA expression of the E3 ubiquitin ligases *MAFbx* and *MuRF1*, increased content of p-GSK-3β, impeding of the eEF2 phosphorylation and as a result maintaining protein elongation at the control level; and (2) high level of phosphorylation of muscle anabolic markers p70S6K and p90RSK.

## 4. Materials and Methods

### 4.1. Ethical Approval

Experiments were performed at the Institute of Biomedical Problems, RAS, Russia. The experiments were approved by the Committee on Bioethics of the Russian Academy of Sciences (protocol 537; 18 February 2020). The study was conducted in accordance with the internationally accepted regulations and rules of biomedical ethics. It is in compliance with the principles and regulations described by Grundy [61].

### 4.2. Animal Procedures

Forty-eight male Wistar rats (three months old, 180–200 g body weight) were randomly assigned to one of the six groups (8 animals per group): non-treated control for 1 and 3 days of experiments (1C and 3C, respectively), 1 and 3 days of unloading/hindlimb suspension (HS) with placebo (1H and 3H, respectively), and 1 and 3 days of HS with PANX1 inhibitor probenecid (PRB) administration (1HP and 3HP, respectively). PRB (Biokanol Pharma GmbH, Germany) was dissolved in PBS at concentration of 20 mg/mL and delivered orally via gavage at a dose of 50 mg/kg of body weight. Rats in control groups received equal amount of placebo solution via gavage. The dose of PRB used in the experiments was established based on the previously published studies [33,62,63,64,65]. In addition, PRB has been approved by FDA since 1979 for the treatment of gout and gouty arthritis. Under normal physiological conditions, muscle pannexin channels are not active and blocking their activity with probenecid (50 mg/kg of body weight) does not change muscle ATP content [65].

Animals were kept at 22 °C in a light-controlled environment (12:12 h light-dark cycle) with unlimited access to water and food. At the completion of one- or three-day experiments rats were euthanized by the intraperitoneal injection of 10% avertin solution at 5 mL/kg of body weight (Sigma-Aldrich Corp., St. Louis, MO, USA). Soleus muscle was dissected, weighed, divided into aliquots, frozen in liquid nitrogen, and stored at −85 °C for the subsequent analyses.

### 4.3. Hindlimb Suspension

Hindlimb suspension was performed using a traction method of noninvasive tail-casting [66]. With this method of unloading rats are free to move around the cage using forelimbs and have food and water ad libitum.

### 4.4. Evaluation of ATP Content

ATP Colorimetric/Fluorometric Assay Kit (MAK190; Sigma, St. Louis, MO, USA) was used for measurements of the ATP content in soleus muscle samples according to the manufacturer’s instructions. In brief, muscle samples were weighted and homogenized with 2N of perchloric acid (PCA; 10 μL per mg of muscle). Samples were kept on ice for 30–45 min and centrifuged at 13,000× *g* for 2 min at 4 °C. The supernatant was transferred into a fresh tube, the volume was measured, and ATP Assay Buffer was added to adjust the volume to 500 μL per mg of muscle tissue. Perchloric acid was neutralized with 2M KOH until the pH reached 6.5–8 and samples were centrifuged at 13,000 × *g* for 2 min at 4 °C. Supernatant was used for ATP quantification. Fifty microliters of supernatant were aliquoted into the plate wells. Fifty microliters of ATP standards (2–10 nmol/well) were also aliquoted into the plate wells. Fifty microliters of ATP reaction mix were added to each well and the plate was incubated for 30 min in the darkness. After that, plates were scanned with Epoch Microplate Spectrophotometer (BioTek, Winooski, VT, USA) at 570 nm.

ATP concentration was evaluated using the following formula:

*ATP concentration = B ∗ DDF/V*

*B*—amount of ATP in the sample well calculated based on the standard curve.
*V*—sample volume added into the wells (50 µL).
*DDF* (deproteinization dilution factor) was calculated using the following formula:
*DDF* = (500 µL + volume KOH (µL))/initial sample volume PCA.

### 4.5. Protein Extraction and Western Blot Analysis

Protein extracts were prepared from frozen soleus muscle. Muscle samples were homogenized in RIPA buffer (#sc-24948, Santa Cruz Biotechnology, Dallas, TX, USA). To protect integrity of the proteins we used Complete Protease Inhibitor Cocktail (#sc-29130, Santa Cruz Biotechnology, Dallas, TX, USA), Phosphatase Inhibitor Cocktail B (#sc-45045, Santa Cruz Biotechnology, Dallas, TX, USA), PMSF (1 mM), aprotinin (10 µg/mL), leupeptin (10 µg/mL), and pepstatin A (10 µg/mL). To evaluate protein concentration in the samples Quick Start Bradford Protein Assay (Bio-Rad Laboratories, Hercules, CA, USA) was used. After dilution in Laemmli buffer samples were run on 10% SDS-PAGE (20 µg/lane) and transferred to a nitrocellulose membrane (Bio-Rad Laboratories, Hercules, CA, USA). Membranes were blocked with blocking buffer (5% nonfat milk powder, TBS pH 7.4, and 0.1% Tween-20) and incubated overnight at 4 °C with the solution of primary antibodies.

The following primary antibodies were used: against total AKT (1:1000; #9272, Cell Signaling Technology, Danvers, MA, USA) and phosphorylated AKT (p-AKT, Ser 473; 1:1500, #4058, Cell Signaling Technology, Danvers, MA, USA), against total p70S6K (1:1000, Cell Signaling Technology, Danvers, MA, USA, #9202) and phosphorylated p70S6K (p-p70S6K; Thr389; 1:2000, Santa Cruz Biotechnology, Dallas, TX, USA, sc-11759), against total p90RSK (1:2000, Cell Signaling Technology, Danvers, MA, USA, #9326) and phosphorylated p90RSK (p-p90RSK; Thr359/Ser363; 1:2000, Cell Signaling, Danvers, MA, USA, #9344), against total GSK-3β (1:1000, Cell Signaling Technology, Danvers, MA, USA, #12456) and phosphorylated GSK-3β (p-GSK-3β; Ser 9; 1:1000, Cell Signaling Technology, Danvers, MA, USA, #9322), against total Erk1/2 (1:1000, Cell Signaling Technology, Danvers, MA, USA, #4695) and phosphorylated Erk1/2 (p-Erk1/2; Thr202/Tyr204; 1:1000, Cell Signaling Technology, Danvers, MA, USA, #9101), against total eEF2 (1:1000, Cell Signaling Technology, Danvers, MA, USA, #2332) and phosphorylated eEF2 (p-eEF2; Thr56; 1:1000, Cell Signaling, USA, #2331), against total AMPK (1:1000, Cell Signaling Technology, Danvers, MA, USA, #2532) and phosphorylated AMPK (p-AMPK; Thr172; 1:1000, Cell Signaling Technology, USA,#2531), against total FOXO3 (1:1000, Cell Signaling Technology, Danvers, MA, USA, #2497) and phosphorylated FOXO3 (p- FOXO3; Ser253; 1:1000, Cell Signaling Technology, Danvers, MA, USA, #9466), and against myogenin (MYOG, 1:500, Invitrogen, Waltham, MA, USA, # MA5-11658).

After three washes with TBS-Tween (TBS and 0.1% Tween-20), the membranes were incubated for one hour with secondary antibodies at room temperature. Horseradish peroxidase-conjugated goat anti-rabbit (1:30,000, #111-035-003, Jackson Immuno Research, West Grove, PA, USA) or goat anti-mouse (1:20,000, #1706516, Bio-Rad, Hercules, CA, USA) secondary antibodies were used. The membranes were washed again in TBS-Tween three times and incubated with Clarity Western ECL Substrate (Bio-Rad Laboratories, Hercules, CA, USA). Blots were evaluated using a C-DiGit Blot Scanner (LI-COR Biotechnology, Lincoln, NE, USA) and Image Studio C-DiGit software. Equal protein loading was verified by membrane staining with Ponceau S (data not shown). The protein expression data for each group are expressed as a percentage of the control group values.

### 4.6. RNA Isolation and Reverse Transcription

Frozen soleus muscle (8–10 mg) was used for isolation of total RNA using RNeasy Micro Kit (Qiagen, Hilden, Germany). Microspin FV-2400 (Biosan, Riga, Latvia) was used for homogenization and centrifugation of the samples. Purified RNA was treated with proteinase K and DNase I, and concentration was evaluated using a NanoPhotometer (Implen GmbH, Munich, Germany). RNA quality of purification was evaluated according to 260/280 and 260/230 ratios. Purified RNA was frozen in aqueous solution at −85 °C for storage. Reverse transcription was performed by incubating 0.5 micrograms of RNA, random hexamers d(N)6, dNTPs, RNase inhibitor, and MMLV (Moloney Murine Leukemia Virus) reverse transcriptase (Moscow, Russia) for 60 min at 42 °C.

### 4.7. Quantitative PCR Analysis

Amplification was performed by mixing one microliter of cDNA with twenty-four microliters of SYBR Green PCR reaction solution containing 1× Quantitect SYBR Green Master Mix (Syntol, Moscow, Russia) and 10 pM of each forward and reverse primer. Sequences of the primers are presented in Table 2. Primers were synthesized by Syntol (Moscow, Russia). Annealing was performed at optimal temperature for each PCR primer-pair. The amplification was monitored using an iQ5 Multicolor Real-Time PCR Detection System (Bio-Rad Laboratories, Hercules, CA, USA). Amplification specificity was confirmed by melting curve analysis. Relative quantification was performed on the basis of the threshold cycle (CT value) for each PCR sample [67]. Initially, two housekeeping genes were evaluated for the normalization: *Gapdh* and *β-actin*. Similar results were obtained when *Gapdh* and *β-actin* were used (data not shown). *Gapdh* was chosen for the normalization of all quantitative PCR analysis experiments in the current study.

### 4.8. Statistical Analysis

PCR data are expressed as median and interquartile range (0.25–0.75). Statistical analysis was performed using REST 2009 v.2.0.12 (Qiagen, Hilden, Germany) and Origin Pro v.8.0 (OriginLab Corp., Northampton, MA, USA) programs. Western blot data are expressed as means ± SE. Significant differences between groups were statistically analyzed using two-way ANOVA followed by Tukey’s test. When normality testing failed, data were analyzed by nonparametric methods (Kruskal–Wallis ANOVA followed by Dunnett’s test). Differences with values of *p* < 0.05 were considered statistically significant.

## 5. Conclusions

Current study for the first time demonstrates that during skeletal muscle unloading PANX1 ATP-permeable channels are involved in the regulation of muscle atrophic processes by modulating expression of E3 ligases and protein translation and elongation processes.

## Figures and Tables

**Table 1:** Soleus Muscle Weight.

Group	Soleus Muscle Weight
1C	89.0 ± 3.8 mg
1H	84.2 ± 1.5 mg
1HP	84.4 ± 2.6 mg
3C	90.6 ± 3.0 mg
3H	73.7 ± 1.7 * mg
3HP	73.6 ± 2.9 * mg

* indicates a significant difference from the control.

**Table 2:** Primers used for QRT-PCR.

Gene	Accession Number	Forward Primer	Reverse Primer
*b-actin*	NM_031144.3	5′-TCATGAAGTGTGACGTTGACATCC-3′	5′-GTAAAACGCAGCTCAGTAACAGTC-3′
*Gapdh*	NM_017008.4	5′-ACGGCAAGTTCAACGGCACAGTCAA-3′	5′-GCTTTCCAGAGGGGCCATCCACA-3′
*MAFbx*	NM_133521.1	5′-CTACGATGTTGCAGCCAAGA-3′	5′-GGCAGTCGAGAAGTCCAGTC-3′
*MuRF1*	NM_080903.2	5′-GCCAATTTGGTGCTTTTTGT-3′	5′-AAATTCAGTCCTCTCCCCGT-3′
*Panx1*	NM_199397.2	5′-CGATGCTGGAGCAGTACTTGAAGA-3′	5′-AGGAGAGGCTGAAGTAGTAGCT-3′

## Data Availability

The experimental data presented in this study are available upon request from the corresponding author.

## Abbreviations

AKT	protein kinase B alpha
AMPK	adenosine monophosphate-activated protein kinase
β-TrCP	beta-transducin repeat containing protein
CaMKII	Ca^2+^/calmodulin-dependent protein kinase II
CARM1	coactivator-associated arginine methyltransferase 1
cdc2	cell division control 2
DHPR	dihydropteridine receptor
GS	glycogen synthase
eEF22	eukaryotic translation elongation factor
eEF2K	eukaryotic translation elongation factor 2 kinase
Erk1/2	extracellular signal-regulated kinase 1/2
FoxO3	forkhead box O3
GSK3β	glycogen synthase kinase-3β
HS	hindlimb suspension
IP3	inositol trisphosphate
MAFbx	ubiquitin ligase muscle atrophy F-box
MYOG	myogenin
MuRF1	muscle-specific RING finger protein 1
mTORC1	mammalian target of rapamycin complex 1
PI3K	phosphatidylinositol 3-kinase
PCr	phosphocreatine
PI3K	phosphatidylinositol 3-kinase
PKA	protein kinase A
PKC	protein kinase C
PANX1	pannexin channel 1
PLC	phospholipase C
p70S6K	70-kDa ribosomal protein S6 kinase
p90RSK90	kDa ribosomal protein S6 kinase 1
PRB	probenecid
P2Y	purinergic G protein-coupled receptor
Rho	Ras Homolog Family Member
RyR	ryanodine receptor
TSC2	tuberous sclerosis complex 2
